# Intravitreal Application: Physicochemical Properties of Drugs Dissolved in Silicone Oils of Different Density in Comparison to the Porcine Vitreous Body

**DOI:** 10.3390/pharmaceutics14071364

**Published:** 2022-06-28

**Authors:** Maximilian Hammer, Sonja K. Schickhardt, Patrick R. Merz, Ramin Khoramnia, Alexander F. Scheuerle, Walter Mier, Philipp Uhl, Gerd U. Auffarth

**Affiliations:** 1 David J Apple Laboratory for Vision Research, 69120 Heidelberg, Germany; maximilianhammer@icloud.com (M.H.); sonja.schickhardt@med.uni-heidelberg.de (S.K.S.); patrick.merz@med.uni-heidelberg.de (P.R.M.); ramin.khoramnia@med.uni-heidelberg.de (R.K.); alexander.scheuerle@med.uni-heidelberg.de (A.F.S.); 2 Department of Nuclear Medicine, Heidelberg University Hospital, 69120 Heidelberg, Germany; walter.mier@med.uni-heidelberg.de (W.M.); philipp.uhl@med.uni-heidelberg.de (P.U.)

**Keywords:** silicone oil, vitreous body, pharmacokinetics

## Abstract

Silicone oil endotamponades provide a reservoir for drugs in the eye. Following vitrectomy surgery to treat retinal detachments, extensive diabetic retinopathy or endophthalmitis, they can be used as long-term lipophilic depots. This study aimed to investigate the physicochemical properties of intravitreally applied drugs of different lipophilicity, namely vancomycin, ceftazidime and voriconazole. For this purpose, an in vitro model of the silicone-oil-filled eye compared to porcine vitreous bodies (PVBs) was used. In a glass container, either light or heavy silicone oil or PVB was set into equilibrium with an aqueous fluid. Vancomycin, voriconazole and ceftazidime were added in concentrations commonly applied in clinical practice. The time course of the concentration of the drugs was determined in the hydrophilic phase for up to 24 h. With silicone oil present, the concentrations of vancomycin, voriconazole and ceftazidime were elevated in the aqueous humor when compared to the vitreous body (*p* < 0.001 for all drugs). With increasing lipophilicity, higher concentrations of the drug dissolved in silicone oil after 24 h (52.7%, 49.1% and 34.3% for vancomycin, ceftazidime and voriconazole, respectively). While no difference between lighter- and heavier-than-water silicone oil was apparent for vancomycin and ceftazidime (*p* = 0.17 and *p* = 0.72), voriconazole dissolved significantly better in the heavier-than-water silicone oil (*p* = 0.002). A higher-than-expected percentage of the glycopeptide vancomycin dissolved in the porcine vitreous body, possibly due to protein binding. In conclusion, silicone oils influence the drug concentration and distribution of intravitreally applied drugs depending on their lipophilicity. The addition of F6H8 used to create heavy silicone oils attenuates these effects for lipophilic drugs. Knowledge of the distribution of these intravitreally applied drugs is crucial to ensure the desired anti-infectious effect.

## 1. Introduction

Since its introduction by Armaly and Cibis in 1962, the use of silicone oil as an intraocular endotamponade has become widespread in vitreoretinal surgery [1,2]. These silicone oils are implanted into the vitreous cavity after the removal of the vitreous body to treat a variety of diseases, most importantly retinal detachments, extensive diabetic retinopathy, endophthalmitis or extensive ocular trauma. Silicone oil has multiple features that warrant its widespread use. In case of retinal detachment or trauma surgery, the effect of silicone oils originates from its high surface tension and lower-than-water density, creating an upward force to reattach the retina to the desired location. On the other hand, in eyes with extensive diabetic retinopathy, silicone oil may “compartmentalize” [3] the eye, and thus can be useful in inhibiting progressive neovascularization through the inhibition of the diffusion of angiogenic substances.

In recent decades, the mechanical and surgical properties of silicone oils have been optimized to deal with the challenges of its use in ophthalmic surgery. Complex handling during surgery has been addressed by adding high-molecular-weight components (HMWCs) with the adaption of the chain length to ease injection while still preserving the favorable properties of high-viscosity silicone oils regarding emulsification. Surgical equipment, such as the cannulas used to inject the oils, have been improved [4,5]. Recently, heavier-than-water silicone oils have been developed to treat inferior pathologies. This is achieved by adding F6H8, a semifluorinated alkane with a high density, to the lighter-than-water silicone oils, which consist of polydimethylsiloxane chains [4,5,6,7,8]. The greater density of these oils creates a downward force to deal with pathologies at the bottom of the eye. While these developments allow for better treatment options in patients with inferior pathologies, the physicochemical properties of these new compound silicone oils are greatly altered but are not well-studied [6].

Pathologies that require the use of silicone oils may come along with intravitreal injections of drugs into the vitreous cavity. Pharmacological aspects related to the major change from the hydrophilic vitreous body to the lipophilic environment of silicone oil have not yet been systematically investigated. Focusing on anti-infectious drugs, commonly administered drugs show a broad spectrum of lipophilicity and size, ranging from the hydrophilic large glycopeptide antibiotic vancomycin to the lipophilic antifungal agent voriconazole. Few studies have proven the changes in the distribution, concentration and elimination of ceftazidime and vancomycin in a low number of macaque eyes filled with lighter-than-water silicone oil compared to regular eyes with intact vitreous bodies [9,10]. However, no data regarding whether these alterations also hold true in eyes filled with heavier-than-water silicone oils are available. In fact, semifluorinated alkanes are well-known lipophilic drug carriers utilized in eye drops [11] to deliver antifungal drugs, such as voriconazole, which are also intravitreally administered in silicone-oil-filled eyes with endophthalmitis. Information on the possible alterations of drug concentration, distribution and clearance, however, could be crucial in preventing harmful concentration spikes, while simultaneously investigating possible effects on half-life and presumed drug clearance through replacement of the aqueous fluid within the eye are necessary to ensure the desired anti-infectious effect.

However, the interaction of the natural vitreous body with drugs, e.g., by protein-binding is still poorly understood [12] and has not been systematically investigated. While the drug binding of drugs of low molecular weight in the vitreous body may only modestly influence drug half-life in the vitreous body [13], this may be different for larger peptide- and protein-based agents [12,14,15].

Thus, in this study we developed an in vitro model of the posterior segment of the eye to investigate the alteration of drug distribution and concentration under controlled conditions in the heavier- or lighter-than-water silicone-oil-filled eye compared to the natural porcine vitreous body with its preserved three-dimensional structure. Three drugs differing in size and lipophilicity commonly used as a treatment for infectious endophthalmitis, namely vancomycin, ceftazidime and voriconazole, were chosen to outline possible characteristics of drug solubility in silicone oil and semifluorinated alkanes. Additionally, a possible decrease in the unbound drug fraction due to protein-binding in the vitreous body was compared between the drugs.

**Figure 1:**
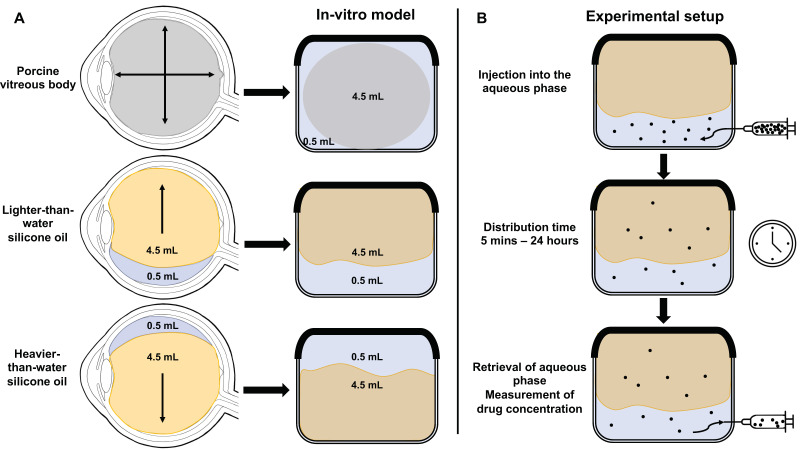
Study design: (**A**) Evaluation of the distribution of three drugs of differing size and lipophilicity in a model of the posterior segment of the eye when exposed to porcine vitreous bodies (top left), lighter-than-water silicone oil (left middle) and heavier-than-water silicone oil (bottom left). (**B**) After injecting the drug into the aqueous phase, it was retrieved after set time points, and the drug concentration was measured in the hydrophilic phase. While the illustration shows the experimental setup for lighter-than-water silicone oil, it was also carried out for heavier-than-water silicone oil and the vitreous body.

Figure 1 presents the setup of the applied in vitro model. In clinical practice, roughly 90% (4.5 mL) of the posterior segment of the eye is filled with silicone oil after vitrectomy surgery, while 10% (0.5 mL) is filled with aqueous humor, a fluid low in electrolytes produced by the ciliary body (Figure 1A). This division was replicated in our model in a glass container (Figure 1B). To allow for a direct comparison between the types of silicone oil and the porcine vitreous body, vitreous bodies were weighed at 4.5 g while preserving their three-dimensional structures and were placed in a glass container with 0.5 mL of balanced salt solution. Three drugs differing in their logP value (a measure for the lipophilicity) were chosen: vancomycin, a large, hydrophilic glycopeptide and voriconazole, a lipophilic antifungal agent, as well as ceftazidime with a lipophilicity ranging between those of vancomycin and voriconazole. The drug concentration was measured in the hydrophilic phase, in a balanced salt solution (BSS). The characteristics of the drugs are presented in Table 1.

## 2. Materials and Methods

### 2.1. Materials

#### 2.1.1. Preparation of the In Vitro Model of the Vitreous Cavity

Porcine vitreous bodies (Schradi Frischfleisch GmbH, Mannheim, Germany) were carefully removed from porcine eyes aged 8–9 months. Vitreous bodies were removed from the vitreous cavity while preserving their original three-dimensional structure. Porcine vitreous bodies of pigs aged 8–9 months have been shown to possess properties comparable to the aged human vitreous body [16]. Subsequently, 4.5 g of the vitreous body was placed in a glass container. Similarly, 4.5 mL of ultra-purified silicone oil with (Densiron 68, Fluoron GmbH, Ulm, Germany) or without the addition of F6H8, a semifluorinated alkane increasing the density of the silicone oil (Siluron 5000, Fluoron GmbH, Ulm, Germany), was used in this study. Vancomycin, voriconazole and ceftazidime were obtained from clinical pharmacies (Vancomycin CP (Hikma Farmacêutica Portugal S.A., Terrugem, Portugal), Voriconalzol (Dr. Friedrich Eberth Arzneimittel GmbH, Ursensollen, Germany), Ceftazidim (Dr. Friedrich Eberth Arzneimittel GmbH, Ursensollen, Germany)).

#### 2.1.2. Preparation of the Hydrophilic Phase with Solution of Voriconazole, Vancomycin and Ceftazidime

A total of 0.5 mL of balanced salt solution was used as an aqueous humor replacement. Voriconazole, vancomycin and ceftazidime were added in concentrations used in daily clinical care. As per clinical protocol, for vancomycin, a dose of 1.0 mg was applied intravitreally in 0.2 mL, thus translating to a starting concentration in the hydrophilic phase of 1.429 mg/mL. A dose of 100 µg of voriconazole in 0.1 mL was intravitreally injected, thus corresponding to a starting concentration of 0.167 mg/mL in the hydrophilic phase. For ceftazidime, 2.0 mg in 0.2 mL was clinically applied, translating to a starting concentration of 2.857 mg/mL.

### 2.2. Methods

#### 2.2.1. Experimental Setup

After the addition of the prepared hydrophilic phase to the vitreous substitute in the glass container, it was placed on a rocking table in a room with controlled temperature at 21 °C and 50% humidity. After 15 min, 30 min, 3 h, 6 h, 12 h and 24 h, the hydrophilic phase was recovered, and the concentration of the respective drug was measured in the hydrophilic phase using high-performance liquid chromatography (HPLC). For experiments with porcine vitreous bodies, additional time points after 5 and 10 min were included. All experiments were carried out sixfold for each time point and for each drug. Figure 1 depicts the experimental setup.

#### 2.2.2. Quantification of Drugs by High-Performance Liquid Chromatography (HPLC)

Quantification of the three drugs (ceftazidime, vancomycin and voriconazole) was performed by reversed-phase HPLC (Agilent 1100 Series) using a C18 column (Chromolith^®^ Performance RP-18e (Merck KGaA, Darmstadt, Germany), 100–3 mm with a linear gradient of 0.1% TFA in water (eluent A) to 0.1% TFA in acetonitrile (eluent B) within 5 min), as previously published for vancomycin and identically applied for ceftazidime and voriconazole [17]. For all 3 drugs, calibration curves, including the concentration ranges of all tested samples with correlation coefficients of >0.995, were established. All drug solutions were retested after 24 h to guarantee stability of the drugs in the respective solutions until quantification by HPLC. Only the hydrophilic phase was analyzed by HPLC.

#### 2.2.3. Statistical Analysis

Data are presented in mean ± standard deviation (SD). Prism Version 8.4.0 (GraphPad Software, San Diego, CA, USA) and STATA 17BE (StataCorp, College Station, TX, USA) were used to analyze data. Normal distribution was examined using Kolmogorov–Smirnov-tests. For comparison, *t*-tests and Mann–Whitney u-tests were used as appropriate.

## 3. Results

### 3.1. Comparison of Drug Distribution between Porcine Vitreous Bodies and Silicone Oils

**Figure 2:**
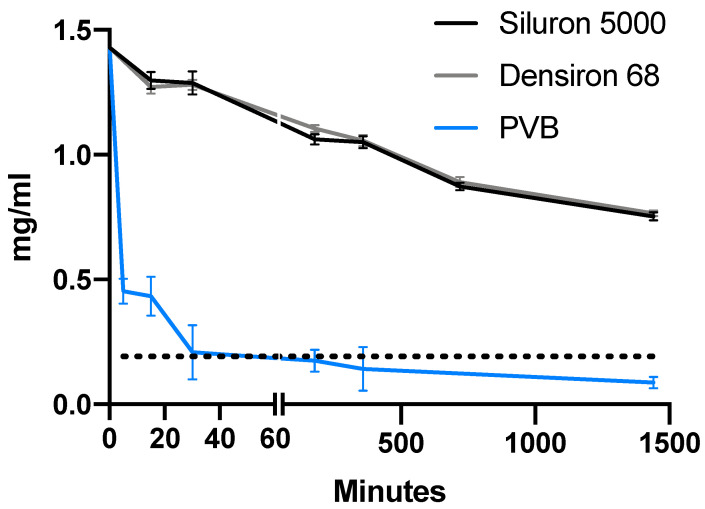
Concentration of vancomycin in the hydrophilic phase in the first 24 h after intravitreal application. Time course of the concentration of vancomycin in the aqueous phase in our model of the silicone-oil-filled eye compared to porcine vitreous bodies. The dotted line depicts the hypothetical concentration for equal distribution between both phases (e.g., aqueous humor and silicone oil). Abbreviations: PVB—porcine vitreous body.

**Figure 3:**
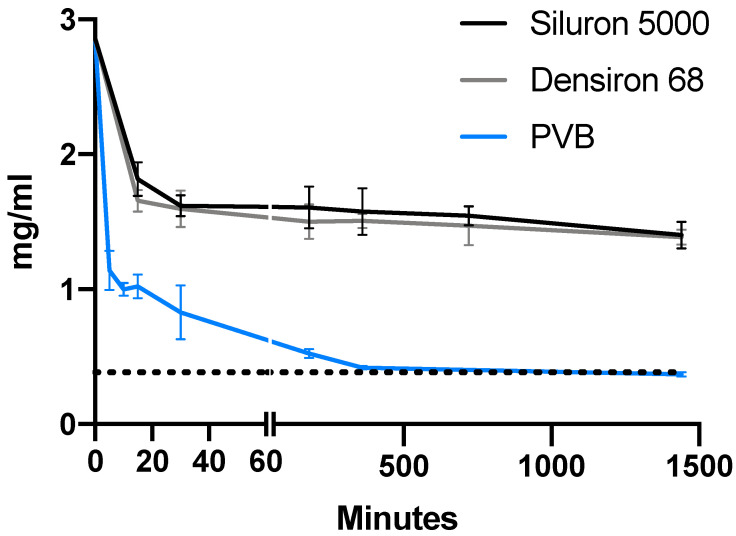
Concentration of ceftazidime in the hydrophilic phase in the first 24 h after intravitreal application. Time course of the concentration of ceftazidime in the aqueous phase in our model of the silicone-oil-filled eye compared to porcine vitreous bodies. The dotted line depicts the hypothetical concentration for equal distribution between both phases (e.g., aqueous humor and silicone oil). Abbreviations: PVB—porcine vitreous body.

**Figure 4:**
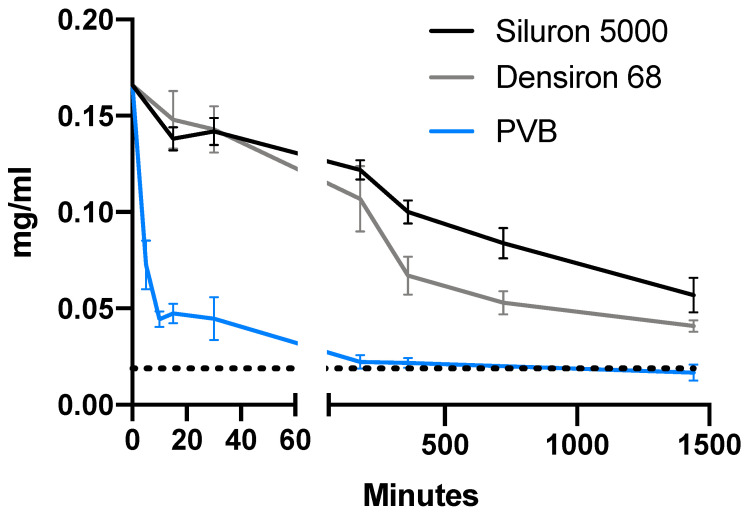
Concentration of voriconazole in the hydrophilic phase in the first 24 h after intravitreal application. Time course of the concentration of voriconazole in the aqueous phase in our model of the silicone-oil-filled eye compared to porcine vitreous bodies. The dotted line depicts the hypothetical concentration for equal distribution between both phases (e.g., aqueous humor and silicone oil). Abbreviations: PVB—porcine vitreous body.

For all three drugs, the concentration in the aqueous phase strongly decreased (ANOVA, *p* < 0.001 for all). Figure 2, Figure 3 and Figure 4 depict the distribution of vancomycin, ceftazidime and voriconazole, respectively. After 24 h, the concentrations in the aqueous phase for all three drugs were increased roughly 8.6-fold (0.75 mg/mL vs. 0.09 mg/mL), 3.8-fold (1.40 mg/mL vs. 0.37 mg/mL) and 3.4-fold (0.057 mg/mL vs. 0.017 mg/mL) for vancomycin, ceftazidime and voriconazole, respectively, when comparing Siluron 5000 to results from the porcine vitreous body model. Similarly, in heavy silicone oil (Polydimethylsiloxane + F6H8), all concentrations were increased in the aqueous phase by a factor of 8.8 (0.77 mg/mL vs. 0.09 mg/mL), 3.8 (1.39 mg/mL vs. 0.37 mg/mL) and 2.4 (0.041 mg/mL vs. 0.017 mg/mL) compared to the aqueous phase of the porcine vitreous body experiments at 24 h.

### 3.2. Differences in Drug Distribution between Light and Heavy Silicone Oil

Interestingly, while no difference at any time point was apparent between the drug concentrations for vancomycin and ceftazidime in lighter- and heavier-than-water silicone oil (*t*-test at 24 h with *p* = 0.17 and *p* = 0.72 (unpaired *t*-tests) for vancomycin and ceftazidime, respectively), voriconazole concentration was lower in the aqueous phase when exposed to heavy silicone oil, indicating a possibly higher dissolvement of voriconazole in the heavy silicone oil (*p* = 0.002, unpaired *t*-test).

### 3.3. Differences in Solubility

**Figure 5:**
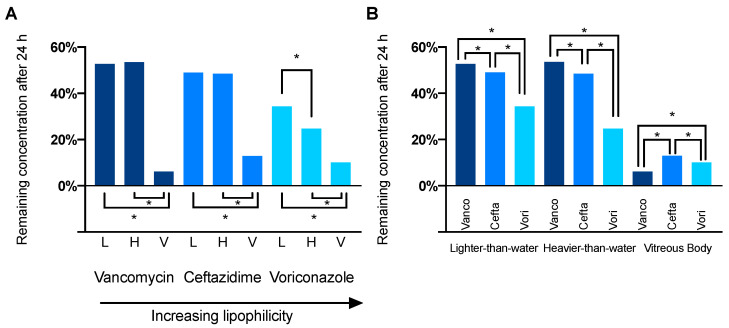
Concentration of vancomycin, ceftazidime and voriconazole in the hydrophilic phase after 24 h in relation to the starting concentration. (**A**) Differences between light and heavy silicone oil were only apparent for voriconazole, the drug with the highest lipophilicity of the 3 studied. All drugs spread best in the porcine vitreous body. (**B**) With increasing lipophilicity (vancomycin < ceftazidime < voriconazole), a greater amount of the drug dissolved in silicone oil. Vancomycin showed greater dissolvement in the porcine vitreous body than both, voriconazole and ceftazidime. Abbreviations: L—lighter-than-water silicone oil, H—heavier-than-water silicone oil, V—porcine vitreous body, * *p* < 0.05.

Figure 5 shows the remaining relative concentration in the aqueous phase for both silicone oil types and porcine vitreous bodies for all tested drugs after 24 h. Interestingly, vancomycin shows a greater dissolvement in the porcine vitreous body than in balanced salt solution, as is also apparent in Figure 2 (theoretical concentration in the aqueous phase with equal dissolvement: 0.192 mg/mL vs. 0.087 ± 0.022 (mean ± SD) mg/mL). After 24 h, the concentration decreased to 52.7, 53.5 and 6.1% for vancomycin, 49.1, 48.5 and 12.9% for ceftazidime and 34.3, 24.7 and 10.1% for voriconazole in lighter- and heavier-than-water silicone oil as well as porcine vitreous bodies, respectively.

## 4. Discussion

In this study, we compared the physicochemical properties of intravitreally applied vancomycin, ceftazidime and voriconazole in a model of the silicone-oil-filled eye with porcine vitreous bodies. Moreover, possible differences between lighter-than-water silicone oil and heavier-than-water silicone oil with the addition of F6H8 were investigated. As the lipophilicity of a drug is crucial for the solubility in silicone oil, we found up to an 8.8-fold increase in drug levels after 24 h compared to our experiments with porcine vitreous bodies. A significant difference between oil types was only apparent for the most lipophilic of the tested drugs: voriconazole.

Few studies deal with the physicochemical properties of intravitreally applied drugs in silicone-oil-filled eyes. Vancomycin and ceftazidime are two of the most frequently used antibiotics to treat infectious endophthalmitis. In 2021, Imamura et al. [9] examined the concentration of both vancomycin and ceftazidime in a small number of silicone-oil-filled, vitrectomized and normal macaque eyes. In line with the data present in our study, levels of both drugs were highly elevated in the aqueous phase harvested from the anterior chamber of the eye after certain time points. As Hira et al. [10] point out in their computational model of the pharmacokinetics for both vancomycin and ceftazidime and Leung et al. [18] in their experimental laboratory study of vancomycin, these elevated levels are most likely caused by the decrease in the volume of distribution in the silicone-oil-filled eye due to the decreased solubility of the drugs in silicone oil compared to the natural vitreous body.

In contrast to all previous studies, we used 5000 cSt silicone oil (Siluron 5000, Fluoron GmbH, Ulm, Germany), which is used as the standard in our clinical practice [6]. While we focused on the distribution of vancomycin and ceftazidime in the first 24 h, the focus of the study conducted by Imamura et al. [9] specifically examines a time frame of up to 10 days after the intravitreal injection. Thus, it remains unclear if the viscosity of the oil, and therefore the movement of the lipophilic and hydrophilic phase in the vitreous cavity, influences the results, especially in the first few hours after the injection of vancomycin and ceftazidime, as higher viscosities could lead to poor distribution within the silicone oil, leading to greater early elimination.

The third commonly intravitreally applied drug we examined in this study was voriconazole. Based on its logP value of 1.82, it shows the highest lipophilicity of all tested drugs in this study (for details on all tested drugs, see Table 1). This is supported by our data, which after 24 h, showed the highest portion dissolved in the lipophilic silicone oil phase compared to vancomycin and ceftazidime (relative concentration in the aqueous phase 34% compared to 53% and 49%, respectively). To our knowledge, this is the first study to ever evaluate the characteristics of voriconazole in a laboratory study of the silicone-oil-filled eye.

During the last decade, a new class of silicone oils were developed by the addition of F6H8 as a compound to silicone oils. With its greater density and high solubility in polydimethylsiloxane, this new generation of silicone oils is heavier-than-water and used to treat inferior pathologies [19,20,21]. All previous studies focused on lighter-than-water silicone oils.

In this study, we also tested the influence of heavier-than-water silicone oil on the distribution of the three previously introduced drugs. While no significant differences for vancomycin and ceftazidime were detected, distinct differences were observed for voriconazole. When using heavier-than-water silicone oil, the concentration of voriconazole was lower in the aqueous phase after 24 h compared to the lighter-than-water silicone oil. The higher solubility of voriconazole in oils containing F6H8 compared to vancomycin and ceftazidime most likely originates from its greater lipophilicity, as reflected by the respective logP values. In fact, F6H8 has not only been used in ophthalmology for the topical application of lipophilic drugs [22,23,24], but also has been researched as a lipophilic drug carrier when aerosolized for inhalative therapy [25] and as a carrier for propofol [26], another highly lipophilic substance. In contrast, polydimethylsiloxane shows a lower solubility for lipophilic drugs: it is difficult to dissolve sufficient amounts of the drug in the oil [27]. This led to researchers modifying polydimethylsiloxane itself to circumvent this by adding copolymers that allow for hydrogen bonding [28,29]. Unfortunately, such chemical modifications lead to higher emulsification rates in vitro, a dangerous complication that can lead to glaucoma and even blindness [30]. In subsequent studies, it should be investigated if further lipophilic drugs, which can be intravitreally applied, show similar tendencies between oil types. While silicone oil thus may be an option to establish a lipophilic drug reservoir in vitrectomized eyes, less invasive strategies must be applied when delivering lipophilic anti-infectious agents to the posterior segment of non-vitrectomized eyes. One possibility is the use of prodrug derivatization. For example, short-chain lipophilic mono-ester prodrugs of ganciclovir showed a greater corneal permeability and significantly increased bioavailability [31,32,33]. Furthermore, other strategies of prodrug derivatization are already in clinical use, such as the dexamethasone implant *Ozurdex^®^* (Abbvie, North Chicago, IL, USA) [34]. In case of no surgical need for the tamponading effect of silicone oil, prodrug derivatization should be preferred to establish drug reservoirs within the vitreous cavity.

Finally, we examined the physicochemical properties of all three drugs in a porcine vitreous body model. The drugs investigated in this study greatly differ in their molecular weight. While their lipophilicity might play a bigger role in their solubility in different kinds of silicone oils, molecular weight and size might be important for their behavior in the vitreous body. Moreover, compared to plasma, the number and concentration of proteins in the vitreous body is significantly smaller [35]. Very little data are available on protein binding in the vitreous body and its influence on the distribution of proteins or peptides [14,15]. Vancomycin, as a glycopeptide, could be influenced by protein binding. Only one previous report examined the binding of antibiotics, including vancomycin, in the human vitreous body. It showed that, with respect to different concentrations, 11 to 42% of vancomycin is protein-bound in the vitreous body after intravitreal application [15]. Again, this is supported by our data. Vancomycin, as by far the drug with the highest molecular weight and the only peptide therapeutic studied in our experiments, showed a drop below the expected concentrations starting 3 h after application, indicating a higher concentration in the vitreous body compared to the aqueous phase, while both voriconazole and ceftazidime equally dissolved in the vitreous and the aqueous phase. Again, these results are in line with Hira et al. [10]. The authors showed that, in vitrectomized monkey eyes compared to eyes with the vitreous body still present, vancomycin is cleared at faster rates, which is possibly caused by the loss of otherwise protein-bound vancomycin that could be slowly released.

While we believe that the goal of this study, which was to investigate the properties of different drugs in the presence of lighter- and heavier-than-water silicone oil compared to the vitreous body, could be achieved by this in vitro model, three limitations should be considered. First, we refrained from the additional implementation of drug clearance as it is present in the living eye through the exchange of the aqueous humor. However, as the clearance is strongly dependent on the volume the drug can dissolve in, a higher clearance can be expected due to the higher drug levels in the aqueous phase. Second, as the measurement of drug concentrations in the silicone oil and vitreous body pose was not applicable using HPLC, we decided to dissolve the drug in the aqueous phase at the beginning of the experiment. While this is close to the real circumstances in silicone-oil-filled eyes due to the injected volume quickly merging with the aqueous phase at the inferior or superior part of the eye due to difference in density, it might not be true for the vitreous body. However, to allow for comparison, we decided to use the same methodology for the vitreous body. Finally, to slow the degradation of the vitreous body, experiments were conducted at 21 °C to slow the possible degradation of the porcine vitreous bodies and to ensure adequate HPLC measurements. While the data (not shown in the manuscript) imply no significant difference in solubility at 37 °C, further studies on in vitro intravitreal drug application should be conducted at body temperature.

## 5. Conclusions

In this study, we present a simply applicable in vitro model to analyze the physicochemical properties of intravitreally applied drugs in eyes filled with silicone oil of different density in comparison to the porcine vitreous body. The differences in density achieved by the addition of semifluorinated alkanes show differentiated effects, specifically for lipophilic drugs. Additionally, possible reservoir functions of the porcine vitreous body due to protein-binding are apparent for vancomycin. Further studies should evaluate other commonly intravitreally applied drugs, such anti-VEGF agents and Amphotericin B, as well as the impact of the viscosity of silicone oil on the distribution of different drugs in the vitreous cavity. This model of the posterior segment of the eye may also allow pre-clinical testing of the performance of new vitreous body replacement strategies with commonly applied drugs [36,37,38,39].

## Figures and Tables

**Table 1:** Molecular weight and logP values were checked on go.drugbank.com (accessed on 22 June 2022); the logP value of ceftazidime is experimental, only logP values of voriconazole and vancomycin are reported.

	Indication	Molecular Weight (g/mol)	LogP
**vancomycin**	Bacterial endophthalmitis	1449.25	−4.4
**ceftazidime**	Bacterial endophthalmitis	546.58	−1.6
**voriconazole**	Fungal endophthalmitis	349.3	1.82

## Data Availability

Data are available from G.U.A. upon reasonable request.
